# Supplementary material for: Bacillamide F, Extracted from Marine Bacillus atrophaeus C89, Preliminary Effects on Leukemia Cell Lines
Source: Biology (Basel). 2022 Nov 25;11(12):1712. doi: 10.3390/biology11121712 (PMC9774924; doi:10.3390/biology11121712)
Supplement: Supplementary file 1 [file biology-11-01712-s001.zip › Table S1.pdf]

Table S1. 1H and 13C NMR (600 and 150 MHz in methanol-d4) data for compound 1.

No.	δ_C, Type	δ_H (*J* in Hz)
1	123.57, CH	7.10, s
2	113.04, C	
3	119.37, CH	7.58, d (7.60)
4	119.65, CH	6.97, t (7.20)
5	122.38, CH	7.07, m
6	112.24, CH	7.32, d (8.00)
7	138.20, C	
8	128.77, C	
9	26.27, CH2	3.05, t (7.20)
10	41.30, CH2	3.68, t (7.20)
11	163.35, C	
12	150.55, C	
13	124.37, CH	8.03, s
14	174.38, C=O	
15	59.66, CH	5.32, dd (2.80, 7.60)
16	33.02, CH2	2.23, m
17	25.12, CH2	2.03, m
18	49.12, CH2	3.71~3.58, m
19	172.64, C=O	
20	22.32, CH3	2.09, s
