# Supplementary material for: Bacillamide F, Extracted from Marine Bacillus atrophaeus C89, Preliminary Effects on Leukemia Cell Lines
Source: Biology (Basel). 2022 Nov 25;11(12):1712. doi: 10.3390/biology11121712 (PMC9774924; doi:10.3390/biology11121712)
Supplement: Supplementary file 1 [file biology-11-01712-s001.zip › Table S2.pdf]

Table S2. IC_{50} values of bacillamide F on different cancer cell lines.

	IC_{50} (μM) 24 h	IC_{50} (μM) 48 h	IC_{50} (μM)72 h
HL60	21.82 ± 2.1	14.13 ± 1.9	9.44 ± 1.6
Jurkat	46.9 ± 2.6	37.16 ± 2.8	33.09 ± 2.3
MRC-5	>100	N.D.	N.D.
PATU8988	>100	N.D.	N.D.
SW 1990	>100	N.D.	N.D.
U937	>100	>100	>100

N.D.= not determine.
