# Supplementary figures and images for: Bacillamide F, Extracted from Marine Bacillus atrophaeus C89, Preliminary Effects on Leukemia Cell Lines
Source: Biology (Basel). 2022 Nov 25;11(12):1712. doi: 10.3390/biology11121712 (PMC9774924; doi:10.3390/biology11121712)

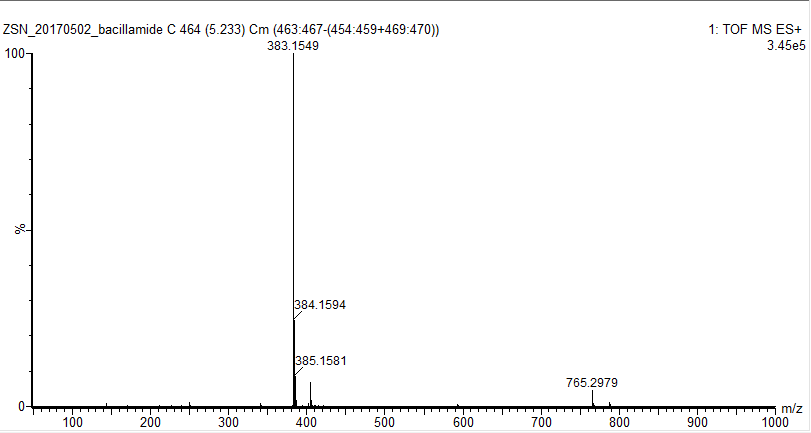

Supplement: Supplementary file 1 [file biology-11-01712-s001.zip › Figure S1.tif]

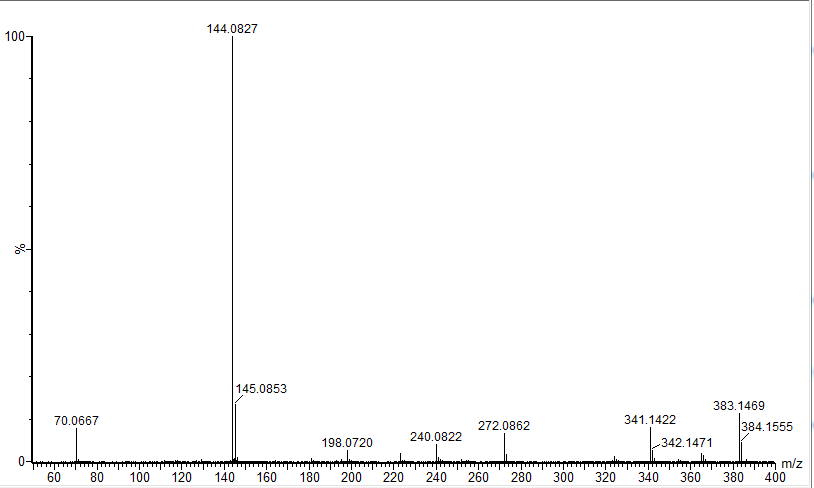

Supplement: Supplementary file 1 [file biology-11-01712-s001.zip › Figure S2.tif]

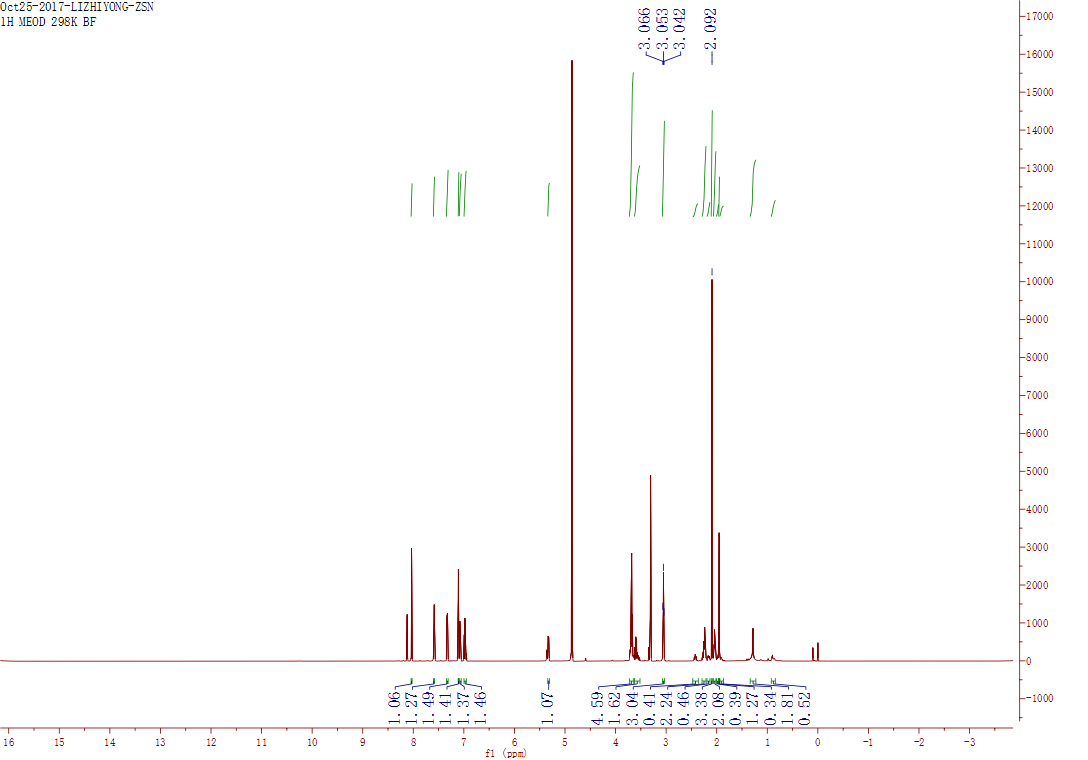

Supplement: Supplementary file 1 [file biology-11-01712-s001.zip › Figure S3.tif]

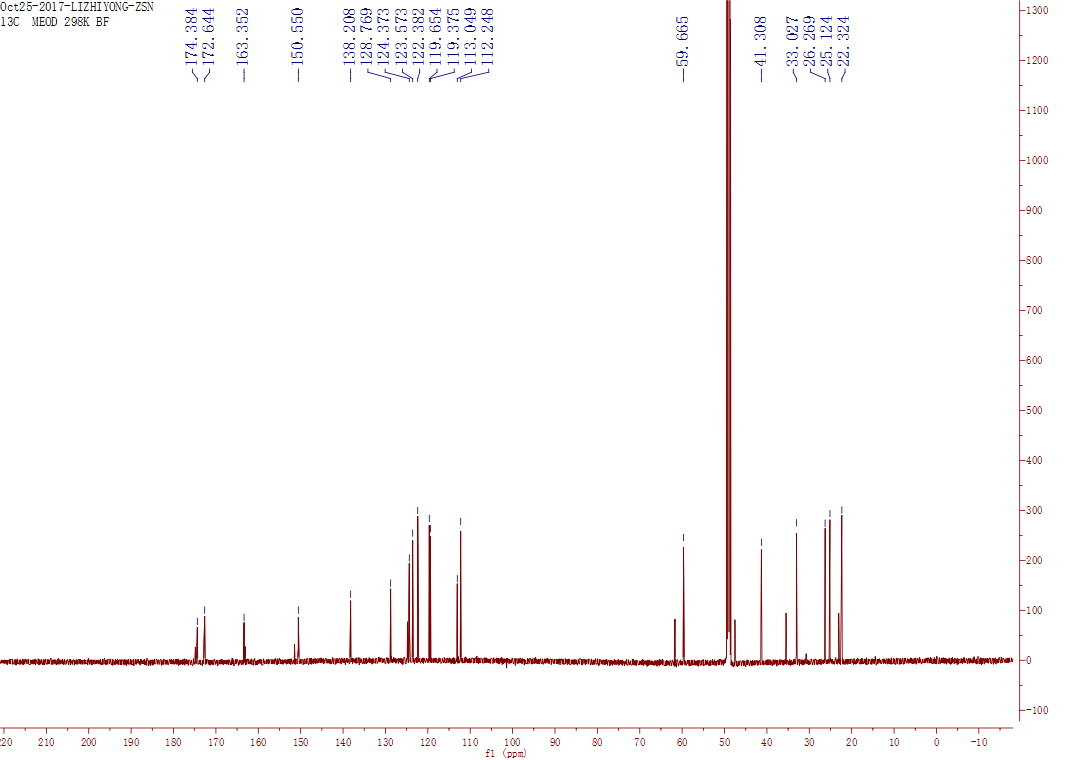

Supplement: Supplementary file 1 [file biology-11-01712-s001.zip › Figure S4.tif]

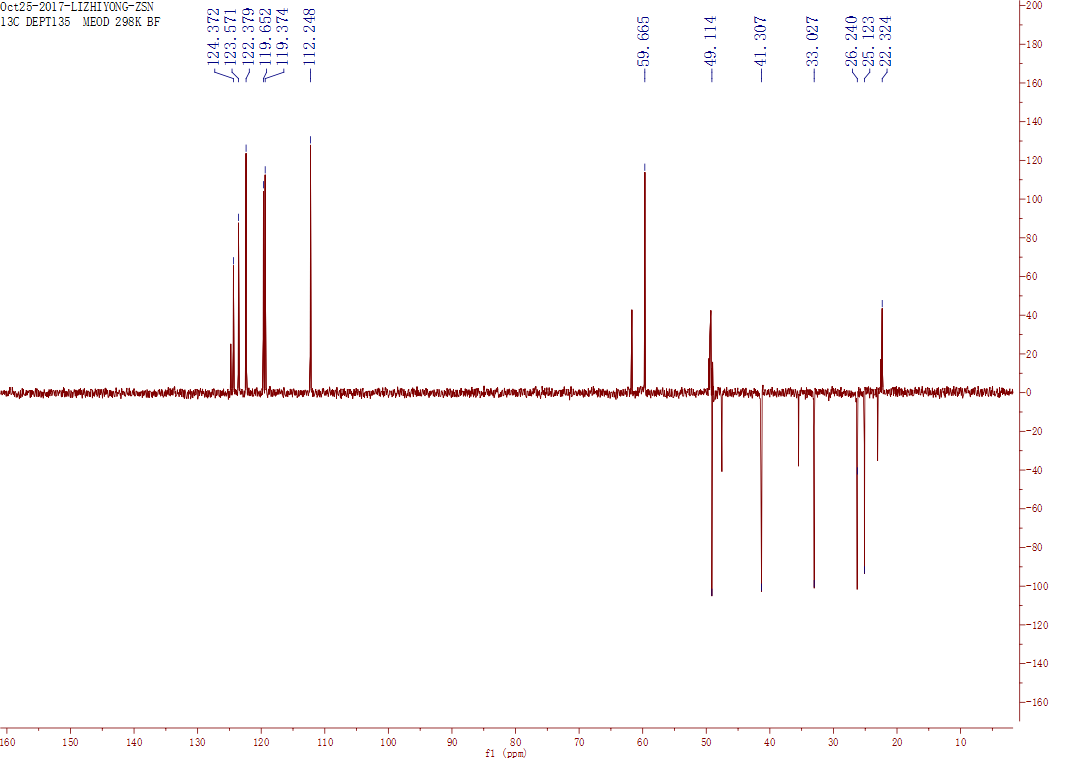

Supplement: Supplementary file 1 [file biology-11-01712-s001.zip › Figure S5.tif]

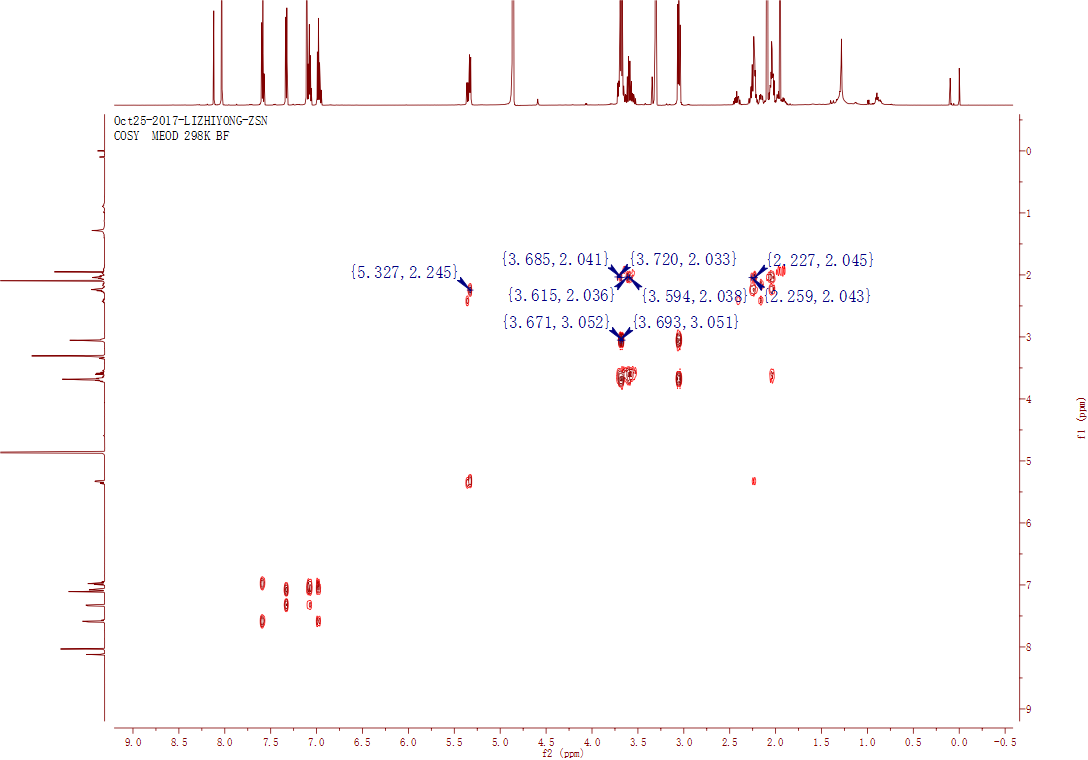

Supplement: Supplementary file 1 [file biology-11-01712-s001.zip › Figure S6.tif]

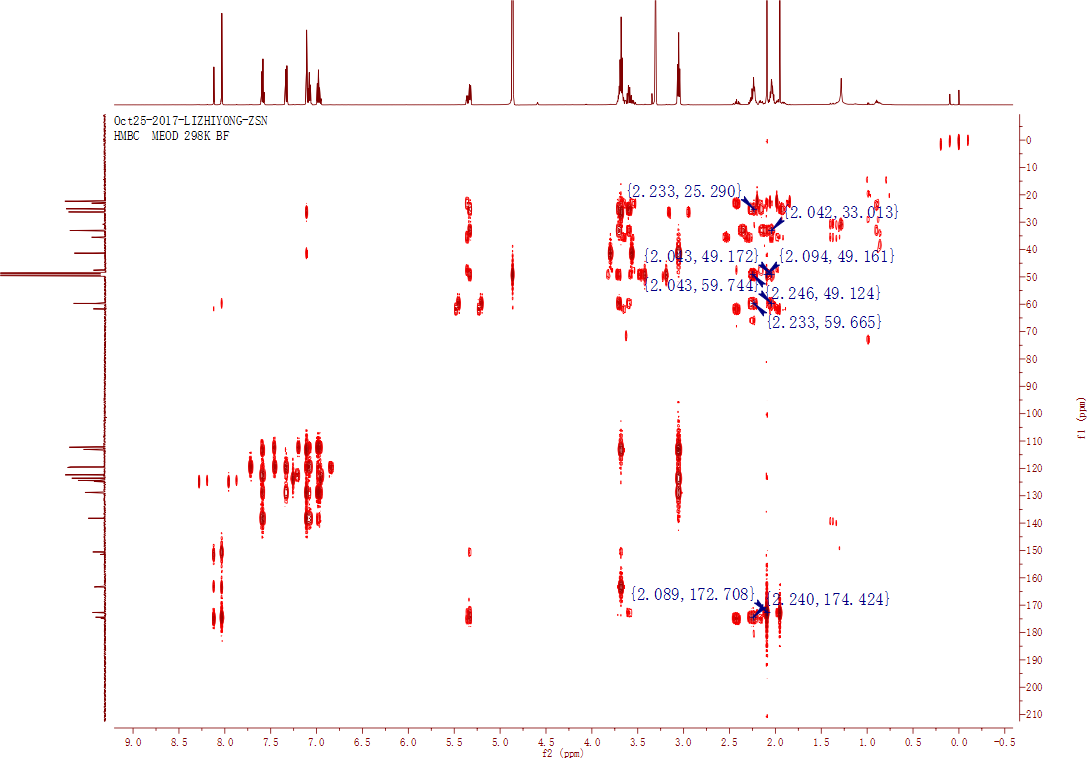

Supplement: Supplementary file 1 [file biology-11-01712-s001.zip › Figure S7.tif]

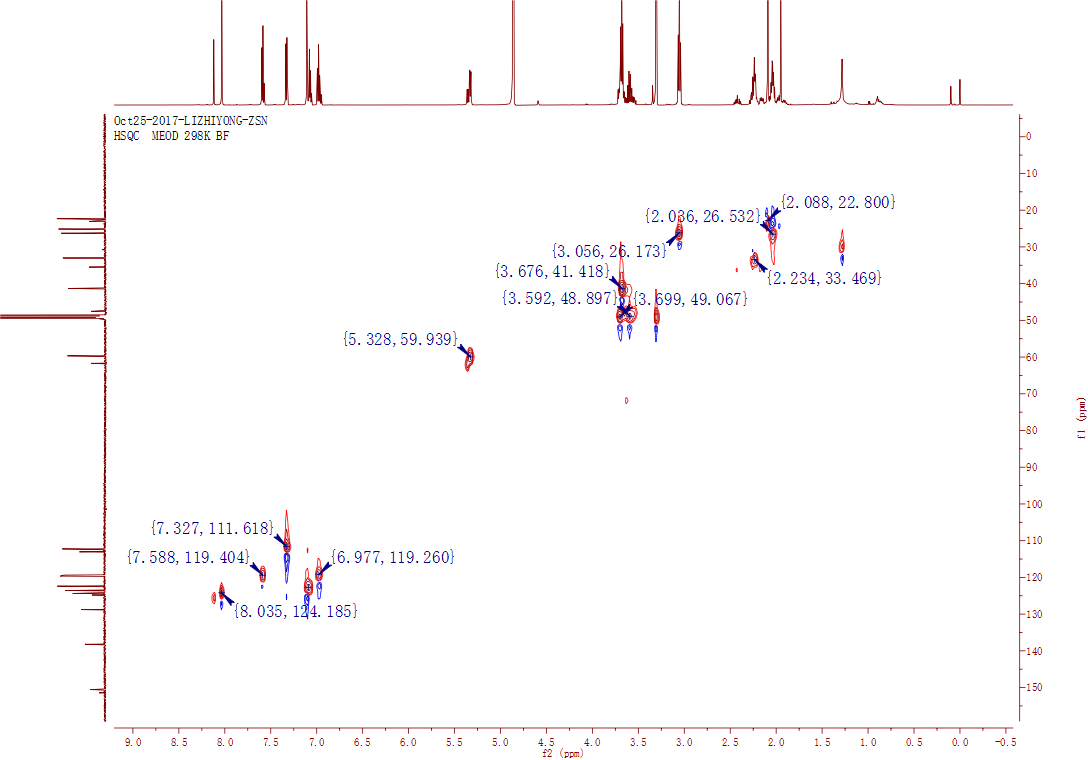

Supplement: Supplementary file 1 [file biology-11-01712-s001.zip › Figure S8.tif]
